# Proteomic profiling of *Sporotrichum thermophile* under the effect of ionic liquids: manifestation of an oxidative stress response

**DOI:** 10.1007/s13205-019-1771-z

**Published:** 2019-05-30

**Authors:** Ayesha Sadaf, Rajeshwari Sinha, Sunil K. Khare

**Affiliations:** Enzyme and Microbial Biochemistry Laboratory, Department of Chemistry, Indian Institute of Technology, Hauz Khas, New Delhi, 110016 India

**Keywords:** Ionic liquids, [EMIM][OAc], [BMIM][BF_4_], *S. thermophile*, Fungal proteome, Anti-oxidative enzymes

## Abstract

*Sporotrichum thermophile,* a known producer of industrial enzymes exhibited stability in the presence of ionic liquids (ILs).The study reports, for the first time, the stress response of *S. thermophile* upon exposure to ILs. In vitro assay showed increased anti-oxidative enzyme levels indicating ROS-mediated oxidative stress by ILs. The proteomic profile and identification of differential proteins confirmed the fungal adaptations by (i) increased expression of glycolytic enzymes and ATP synthases (ii) downregulation of TCA cycle and protein synthesis machinery components (iii) expression of HSP70 and catalase/peroxidase. These changes are indicative of metabolic regulation of many important pathways and how ILs can be used to manipulate protein behavior.

**Electronic supplementary material:**

The online version of this article (10.1007/s13205-019-1771-z) contains supplementary material, which is available to authorized users.

## Introduction

Ionic liquids (ILs) constitute a new class of solvents with a promising future in the field of green technology (Smiglak et al. 2014). These solvents, organic salts by nature, exhibit unique properties like negligible vapor pressure, high solvating ability and thermal stability. They simultaneously offer the advantage of being easily modifiable, which confers on them different properties based on the cation and anion modified. These have, hence, been termed as “designer solvents” (Dixit 2013).

The exploitation of ILs is majorly in the area of biomass processing for the generation of second-generation biofuels (Hou et al. 2017; Sheldon 2016). The lignocellulosic biomass is quite recalcitrant in nature due to the presence of cellulose component. Hence, it is pre-treated with ILs in the first step to loosen it or to make it accessible for enzyme-mediated saccharification in the subsequent step (Khare et al. 2015). Since ILs have shown to exert toxic effect towards saccharifying enzymes like cellulases and xylanases, the IL-pre-treated biomass has to be washed with water several times to remove the residual IL (Yu et al. 2016). In this context, the present approaches deal with the screening or engineering of enzymes which are stable in the presence of ILs so that the whole process can be carried out in a single pot (Xu et al. 2016). Due to the high cost of enzymes, biocatalysis using microbial cells has recently gained momentum, since it is cost effective and maintains a native domain for functioning of enzymes (de Carvalho 2017). On similar lines, if the microbial cells are stable towards the ILs effect, the enzymes produced by them will also be active under such conditions thereby enabling the development of an in situ pre-treatment and saccharification approach. The suitability of ILs for biocatalytic whole-cell applications is gaining momentum but the studies pertaining to it are quite limited due to toxic effects of ILs on many microorganisms (Shen et al. 2016). Generally the conventional types of ILs are quite damaging to the bacterial cells causing membrane perturbance and lysis (Bhattacharya et al. 2018). However, molecular level understanding on the interaction of ILs with bacteria, yeast or fungus is presently limited.

*S. thermophile* (syn. *Myceliophthora thermophile*) is known to produce a number of industrially relevant enzymes such as xylanases, cellulases, phytases, esterases and pectinases (Singh 2016). To further explore the usefulness of using *S. thermophile* cells for IL-mediated saccharification, their stability in ILs has been previously studied (Sadaf and Khare 2017). In this work, it was observed that *S. thermophile* was able to grow profusely and produce xylanase in the presence of two most commonly used ILs, [EMIM][OAc] and [BMIM][MeSO_4_], indicating high tolerance of the fungus towards these ILs. The IL stability of these adapted cultures further led to cell-based simultaneous pre-treatment and saccharification.

A mechanistic understanding of the effects of ILs on fungal cells can help to explain such stability and outline future possibilities of enhancing biotransformation yield. Considering this, *Sporotrichum thermophile* was selected to further study IL-induced stress responses upon exposure to a compatible IL, [EMIM][OAc] which did not majorly affect *S. thermophile* growth and xylanase production and a highly non-compatible IL, [BMIM][BF_4_], which drastically inhibited *S. thermophile* growth and xylanase production. Hence, the anti-oxidative enzyme levels and global proteomic changes in *S. thermophile* were studied under the influence of these two ILs. With few proteomic studies on fungal systems reported, the current work is relevant since proteomic analysis on IL interaction with an extremophilic fungus is being reported for the first time. An interpretation of the fungal behavior upon IL exposure will help in expanding the knowledge on IL induced stress as well as its adaptations. Results from this study will also help to reflect on how such unique solvents can be used in future to manipulate proteins of biotechnological relevance.

## Materials and methods

### Materials

1-Butyl-3-methylimidazolium tetrafluoroborate [BMIM][BF_4_] and 1-ethyl-3-methylimidazolium-acetate [EMIM][OAc] were purchased from Sigma-Aldrich (St. Louis, MO, USA). All other chemicals used were of analytical grade.

### Microorganism and growth conditions

*S. thermophile* isolate was provided by Prof. T. Satyanarayana, Department of Microbiology, Delhi University South Campus, New Delhi, India. The mould was maintained on Potato Dextrose Agar (PDA) slants and subcultured at 15-days interval. Preparation of the inoculum was carried out as per the method described earlier (Sadaf et al. 2016). Briefly, a loopful of fresh mycelium (10.0 mg) was taken from a PDA slant and suspended in 5.0 ml of saline to form a spore suspension. This inoculum was added at 2.0% (v/v) concentration in Emerson’s medium containing (g/L): soluble starch 15.0, yeast extract 4.0, K_2_HPO_4_ 1.5, MgSO_4_ 0.5, pH 9.0. Incubation was done at 40 °C for 24 h.

*S. thermopile* growth and xylanase production in the presence of IL was previously studied in Emerson’s medium (Sadaf and Khare 2017).

### Estimation of anti-oxidative enzyme activities

Erlenmeyer flasks (50.0 mL) containing 10.0-mL Emerson’s medium and varying concentrations of [EMIM][OAc], [BMIM][BF_4_] (according to their compatibility levels with *S. thermophile*) were seeded with 2.0% (v/v) inoculum of 24-h grown culture. This was followed by incubation of the setups at 40 °C and 100 rpm for varying time periods (24–72 h). In each case, entire flask was harvested and centrifuged at 10,000×*g* for 10 min. The mycelia were washed twice with phosphate buffer (50.0 mM, pH 7.0) and their suspension in 1.0 mL of same buffer was sonicated (1–2 min, cycle of 40%, frequency of 20 kHz) at 4 °C. Mycelial lysates were centrifuged (8000×*g*; 10 min; 4 °C) to remove cell debris and supernatant was used for estimation of anti-oxidative enzyme activities viz. catalase and glutathione reductase. A control, without any IL, was also run in parallel.

Catalase activity was estimated by the method of Aebi (1984). One unit is defined as the millimoles of H_2_O_2_ reduced by the enzyme per minute under standard assay conditions (pH 7.0, 25 °C). Glutathione reductase assay was performed as per the method of Mavis and Stellwagen (1968). The calculations were based on the molar extinction coefficient of H_2_O_2_ at 240 nm (43.6 M^−1^ cm^−1^). One unit of glutathione reductase activity is defined as the amount of enzyme required to catalyze the reduction of one micromole of oxidized glutathione (GSSG) to reduced glutathione (GSSGH) per minute under standard assay conditions as mentioned above. Calculations were based on ε of NADPH (6200 M^−1^ cm^−1^).

### Sample preparation for 2D gel electrophoresis

*S. thermophile* was grown in Emerson’s medium in the absence and presence of 0.5% (v/v) of ILs, [EMIM][OAc] and [BMIM][BF_4_]. The mycelium obtained after 24 h was processed to obtain the intracellular proteome sample using modified method of Carpentier et al. (2005). Briefly, the harvested mycelia (control and ILs treated cultures) were first washed with phosphate buffer saline (pH 7.4). Lysis was performed by grinding the washed mycelia in liquid N_2_ and suspending them in chilled lysis buffer (0.02-M Tris–HCl, pH 8.5) containing 25.0-mM NaCl, 2.0-mM EDTA, 10.0% (v/v) glycerol, 0.5% (v/v) Triton X-100, 10.0-mM 2-mercaptoethanol, 1.0-mM DTT, 1.0-mM PMSF, 10.0-mM NaF. After sonication (2 × cycles each of 40%, 120 W, 20 kHz for 5 min) on a Biologics Ultrasonic Homogenizer (Virginia, USA), the mycelial lysates were centrifuged to remove cell debris and the protein content in supernatant was precipitated using 10.0% (v/v) ice cold TCA for 45 min followed by acetone washing (3–4 washes each for 10 min, centrifuged at 6000×*g*, 4 °C). Protein estimation was done using Bradford method (Bradford 1976). The protein samples were dissolved in 500.0-µl solubilization buffer (8.0 M urea, 4.0% (w/v) CHAPS, 1% (w/v) DTT, De-Streak reagent (Plus One, GE Healthcare) and loaded onto non-linear pH 3–10 IPG strips (125.0 µl sample containing 250.0 µg of protein, 150.0 µl rehydration buffer and 2.0% v/v IPG buffer, containing pharmalytes in the range of 3–10) for overnight rehydration.

### Isoelectric focusing, 2D Gel electrophoresis and spot analysis

Isoelectric focusing (IEF) was performed on an Ettan IPGphor 3 instrument (GE Healthcare, Buckinghamshire, UK) for 12,000 Volt-hour (Vh). Following IEF, the IPG strips were incubated in the SDS equilibration buffer (6.0-M urea, 75.0-mM Tris–HCl at pH 8.8, 30.0% (v/v) glycerol, 2.0% (w/v) SDS, 0.002% (w/v) bromophenol blue containing 1.0% (w/v), dithiothreitol) for 15 min. The second incubation was again carried out in the same buffer containing 2.5% (w/v) iodoacetamide for 15 min. For second-dimension SDS-PAGE, strips were transferred on 12.5% polyacrylamide gels and electrophoresed (Mini PROTEAN gel electrophoresis unit (BioRad Laboratories, USA). Gels were stained with Coomassie Brilliant Blue R-250 and destained using methanol: acetic acid: water solution (30:10:60). Triplicate gels were run for each of the sample prepared. All gels were scanned in BioRad Gel documentation system (BioRad Laboratories, USA). Gel images were analyzed using ImageMaster Platinum 7 software (GE Health Care). Spots showing a differential expression with minimum 1.5-fold change in expression levels were considered for further analysis.

The selected spots were processed for in-gel tryptic digestion (Sigma-Aldrich in-gel tryptic digestion kit, SigmaAldrich, St. Louis, USA) as per manufacturer’s instruction. Briefly, the digested peptides were eluted on a 2.2 µm, 120° A (2.1 × 150 mm) Dionex column (Acclaim TM RSLC C18) using LC (Dionex Ultimate 3000) (Thermo Scientific, Geel, Belgium). The mobile phase consisted of solvent A (0.1% v/v, formic acid in water) and solvent B (0.1% v/v, formic acid in ACN). Sample (40 µl) was injected and the linear gradient was started with B set to reach from 10 to 75% (v/v) in 25 min, increased further from 75 to 90% (v/v) in 10 min, held at 90% (v/v) for 10 more min and subsequently reducing to 10.0% (v/v) in 10 min. The column effluent was connected to an ESI nano-sprayer on microTOF-QII (Bruker, Bremen, Germany). The raw mass data obtained were analyzed with the help of Compass Data Analysis software which generated a mascot generic file format which was processed for peak list generation using the Mascot 2.4.01 search engine (Matrix Science, London, UK) through the BioTools software version 3.0 (Bruker Daltonics, Billerica, USA). The Mascot search was performed by setting taxonomy to ‘All entries’ and NCBInr or SwissProt were used for different searches. Enzyme trypsin was selected from the list, partial (cleavages per peptide) was set to 1, carbamidomethyl (C) was set to a global modification and the variable modification was set to Oxidation (M). The mono-isotopic peptide mass tolerance was set from 25 to 75 ppm depending on the sample, while the fragment ion mass tolerance was set to 0.2 Da. Mascot scores above the zone of significance were taken to be valid for the protein identity.

Since the genome of *S. thermophile* (syn. *Myceliophthora thermophila*) has not been completely annotated, hence the top peptide matches were blasted to a nearest phylogenetic member protein. All the experiments in this work were performed in triplicates and variation was within ± 5%.

## Results and discussion

### Effect on anti-oxidative enzyme activities of *S. thermophile* on exposure to ionic liquids

**Fig. 1:**
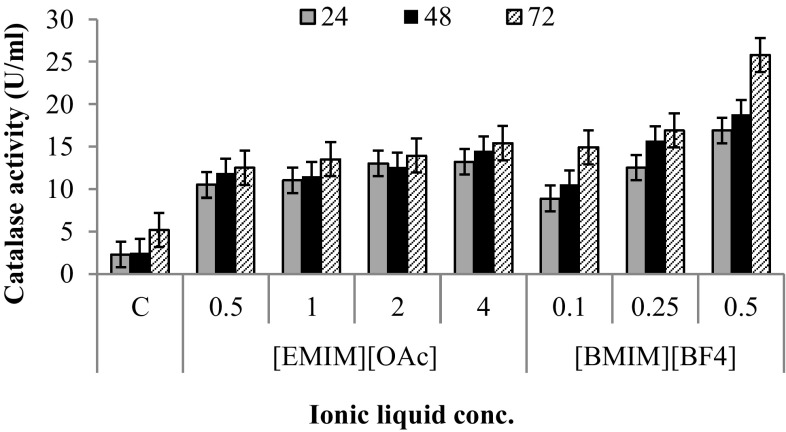
Catalase activity in the presence of varying concentration of ionic liquids (0.25, 0.5, 1.0, 2.0% (v/v) for [EMIM][OAc]; 0.1, 0.25, 0.5% (v/v) for [BMIM][BF_4_]). 24 h (  ); 48 h (  ); 72 h (  )

*S. thermophile* was grown in Emerson’s medium in the presence and absence of varying concentration of ILs ([EMIM][OAc] and [BMIM][BF_4_]). The effect of ILs was examined on resultant catalase activity levels (Fig. 1). In case of [EMIM][OAc], the maximum catalase activity (15.0 U/ml) was recorded at 4% (v/v) [EMIM][OAc] as compared to 5.0 U/mL in the control (absence of IL). The enhancement was much higher (26.0 U/ml) in 0.5%, (v/v) concentration of [BMIM][BF_4_] indicating major oxidative damage by this IL.

Similar trend was observed for glutathione reductase activity which was found to be highest at 4.0% (v/v) [EMIM][OAc] (15.0 U/mL as compared to 6.0 U/mL in control) after 72 h (data not shown). The [BMIM][BF_4_] caused much higher effect leading to 35.0 U/mL activity at 0.5% (v/v) concentration. Overall, [BMIM][BF_4_] caused an increased response and higher anti-oxidative enzyme levels as compared to [EMIM][OAc]. This indicates that [EMIM][OAc] may not have caused severe oxidative damage to *S. thermophile* cells, whereas [BMIM][BF_4_] drastically affected them and led to more pronounced anti-oxidative enzyme production. Stress-induced production of anti-oxidative enzymes in microbial cells play a vital role in scavenging the reactive oxygen species (ROS) to prevent oxidative cell damage (Zhao and Drlica 2014). Further, proteomic studies were carried out to probe the changes in the intracellular protein expression as a result of exposure/possible stress caused by ILs. The effect of ILs on the proteomic profile of fungi has not been explored in much detail earlier. A previous study performed on *Aspergillus nidulans* and *Neurospora crassa* showed the alteration in the synthesis of secondary metabolites and biochemical pathways under the effect of 1-ethyl-3-methylimidazolium chloride and cholinium chloride (Martins et al. 2013).

### Proteomic profile of *S. thermophile* in the presence and absence of ILs

**Fig. 2:**
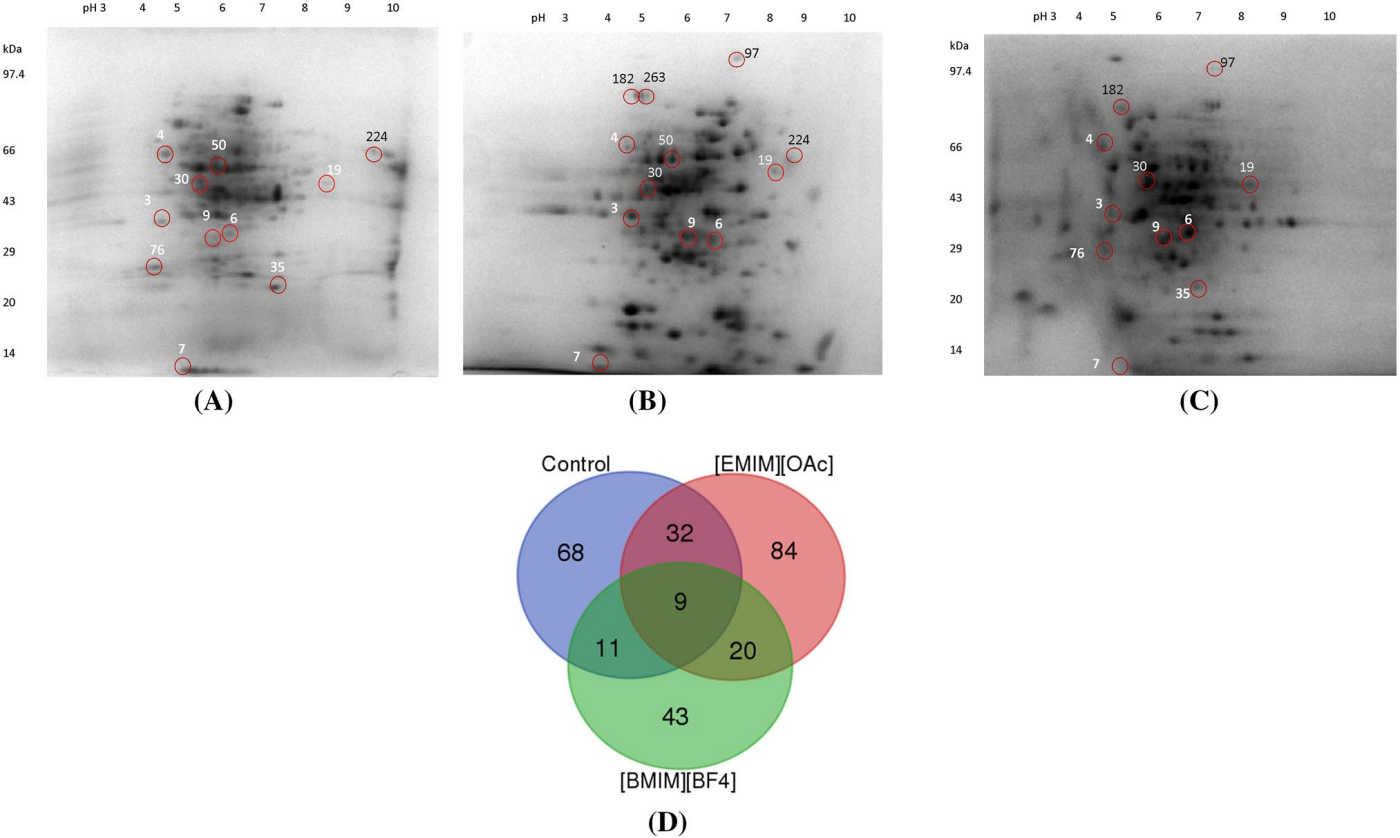
Two-dimensional gel electrophoresis analysis of the intracellular proteome of *S. thermophile* in the presence and absence of ILs. **a** Control (in the absence of ILs); **b** In the presence of 0.5% (v/v) [EMIM][OAc]; **c** In the presence of 0.5% (v/v) [BMIM][BF_4_]; **d** Venn diagram representing the overlapping of proteins between the control and IL-treated *S. thermophile* cells

The proteome map of *S. thermophile* mycelium obtained for control, [EMIM][OAc] and [BMIM][BF_4_] treated samples is shown in Fig. 2a–c. Distinct global changes were observed in the IL-treated gels. While many proteins were differentially expressed, some new proteins were further observed to be expressed only in response to ILs. Venn diagram in Fig. 2d shows that expression of 68 spots was suppressed in the presence of both ILs. While 84 and 43 spots remained exclusive to each [EMIM][OAc] and [BMIM][BF_4_], respectively, twenty spots were common to both. On the other hand, 32 spots were commonly expressed in [EMIM][OAc] and control, whereas only 11 spots were shared between [BMIM][BF_4_] and control. The differential expression of proteins in control as well as in all the ILs exposed cells was highly significant in 9 spots. Among these, spots 1, 7, and 10 were downregulated; 6, 9, 19 and 30 were upregulated in the presence of [EMIM][OAc] and [BMIM][BF_4_]. Spot 3 was upregulated in [EMIM][OAc] and downregulated in [BMIM][BF_4_]; whereas spot 4 was downregulated in [EMIM][OAc] and upregulated in [BMIM][BF_4_] (Fig. 2a–c).

### Identification/characterization and functional co-relation of differentially expressed proteins in response to ILs

The prominent spots which appeared commonly in the control and IL-treated samples were further analyzed by ESI MS–MS.

The theoretical pI of all the identified proteins and their molecular weights ranged from 4.17–9.3 and 11.3–82.0 kDa, respectively. The identity, characteristics and function of identified proteins are summarized in Table 1. The identified proteins belonged to many enzymes of the central carbon metabolism such as glycolysis, TCA cycle and pentose phosphate pathway (Supplementary Fig. 1). Spots Nos. 6 and 30 were found to be upregulated in both the ILs. These spots matched with glyceraldehyde 3-phosphate dehydrogenase (GPD) and enolase respectively which are important glycolytic pathway enzymes (Kim and Dang 2005). An increase in the expression of GPD has been previously reported in stress conditions like oxidative and citric acid stress as well as in responses to osmoadaptations in epithelial cells, *Saccharomyces cerevisiae*, *Lactobacillus rhamnosus* as well in *A. nidulans* (Kim et al. 2006). This indicates that this pathway has been enhanced under IL-stress, suggesting a possible survival strategy under stress conditions (Wu and Wei 2012). Spot No. 9 which was also upregulated matched with transaldolase, an enzyme involved in the oxidative phase of the pentose phosphate pathway. The expression of this enzyme has been shown to increase in early oxidative stress conditions (de Arruda et al. 2013). The upregulation of this pathway under oxidative stress conditions generates NADPH which plays a defensive role against oxidative damage (Voodisch et al. 2011). Another upregulated spot in both ILs (Spot No. 19) was identified as isocitrate dehydrogenase which is a citric acid/TCA cycle enzyme. The NADP-dependent isocitrate dehydrogenase catalyses the formation of α-ketoglutarate, a key intermediate involved in ROS regulation (Goncalves et al. 2012; Mailloux et al. 2007). The expression of pentose phosphate pathway proteins as well as increased levels of isocitrate dehydrogenase enzyme have been shown to increase majorly in response to various types of oxidative stress conditions like temperature, osmotic, pH, etc. to generate reducing power (Tomanek 2015). The levels of ATP synthase and mitochondrial ATP synthase corresponding to Spots Nos. 50 and 224, respectively, increased only in the presence of [EMIM][OAc] indicating greater energy requirements (Soini et al. 2005). Downregulated spots No. 1 and 10 (not visible in gel images) in both ILs were identified as malate dehydrogenase and citrate synthase, respectively. A drop in expression levels of TCA cycle enzymes probably explains the slowing down of TCA cycle. Similar results have been observed in the case of *Paracoccidioides* yeast cells coping under oxidative stress induced by H_2_O_2_ (de Arruda et al. 2013). Spot no. 7 was identified as 60S ribosomal protein and was downregulated in both the ILs. Similarly, spots nos. 76 and 35 were identified as, EF-1β and nucleotide diphosphate kinase, respectively, and were downregulated only in [BMIM][BF_4_] indicating retardation of protein synthesis machinery. In environment stress conditions, the lowering of protein synthesis has earlier been implicated in the reduction of ROS, since this process leads to generation of H_2_O_2_ (Tomanek, 2012). Spots Nos. 182, 263, 97 pertaining to newly developed spots generated exclusively in the presence of ILs were identified as HSP70 and catalase/peroxidase. Heat shock proteins have also been reported to be expressed in *Paracoccidioides yeast* cells exposed to H_2_O_2_-induced oxidative stress conditions (de Arruda et al. 2013). While HSP70 is a molecular chaperone responsible for maintaining the correct folding of proteins under stress conditions, catalase/peroxidase proteins are anti-oxidative enzymes which protect cell from ROS-induced damage (de Arruda et al. 2013). Therefore, the above results indicate the induction of oxidative stress on exposure to ILs.

**Table 1:** Characteristics and function of intracellular proteins affected in *S. thermophile* grown in presence of ionic liquids

Protein spot No.	Protein name	Expression level	NCBI accession number	Mol wt. (Da)	pI	Mascot score	Sequence coverage	No. of matched peptides	Function	References
A. Protein spots present in control, [EMIM][OAc], [BMIM][BF_4_]										
6	Glyceraldehyde 3 phosphate dehydrogenase	Upregulated in both ILs	gi|367035068	36462	6.54	402	29%	5	Catalyzes sixth step of glycolysis	Kim and Dang (2005)
9	Transaldolase	Upregulated in both ILs	gi|367021512	35278	5.65	69	4%	10	Transaldolase connects the pentose phosphate pathway to glycolysis.	de Arruda et al. (2013)
19	Isocitrate dehydrogenase	Upregulated in both ILs	gi|367030375	52569	8.74	333	17%	5	Catalyses the oxidative decarboxylation of isocitrate into alpha-ketoglutarate in the TCA cycle	(Mailloux et al. 2007)
30	Enolase	Upregulated in both ILs	gi|367019896	47705	5.27	711	38%	14	Responsible for the catalysis of the conversion of 2-phosphoglycerate (2-PG) to phosphoenolpyruvate (PEP), in the penultimate step of glycolysis	Kim and Dang (2005)
1	Malate dehydrogenase	Down-regulated in both ILs	gi|367028550	35386	8.67	464	27%	7	Reversibly catalyses the oxidation of malate to oxaloacetate	Mailloux et al. (2007)
7	60S ribosomal protein	Down-regulated in both ILs	gi|367026083	11295	4.17	112	47%	2	Component of ribosome	Shenton et al. (2006)
10	Citrate synthase	Down-regulated in both ILs	gi|116204913	52760	8.54	117	5%		Catalyses the first reaction of the citric acid cycle (TCA cycle)	Mailloux et al. (2007)
3	ND									
4	ND									

Protein spot No.	Protein name	Expression level	NCBI accession number	Mol wt. (Da)	pI	Mascot score	Sequence coverage	No. of matched peptides	Function	References
B. Protein spots present only in control and [EMIM][OAc]										
50	ATP synthase	Upregulated in [EMIM][OAc]	gi|367035526	55576	5.33	915	40%	9	Synthesizes ATP from ADP and inorganic	Soini et al. (2005)
224	Mitochondrial ATP synthase	Upregulated in [EMIM][OAc]	gi|367019566	59626	9.3	232	10%	6	Catalyses the synthesis of ATP	Soini et al. (2005)

Protein spot No.	Protein name	Expression level	NCBI accession number	Mol wt. (Da)	pI	Mascot score	Sequence coverage	No. of matched peptides	Function	References
C. Protein spots present only in control and [BMIM][BF_4_]										
76	EF 1β	Down-regulated in [BMIM] [BF_4_]	gi|367032192	25085	4.39	276	20%	3	Its involved in the transfer of aminoacylated tRNAs to the ribosome	Shenton et al. (2006)
35	Nucleotide diphosphate kinase (NDPK)	Down-regulated in [BMIM] [BF_4_]	gi|367024509	16775	6.85	75	11%	17	(i)Phosphotransferring activity from mainly ATP to NDPs generating nucleoside triphosphates (NTPs) (ii) Autophosphorylation activity from ATP and GTP	Shenton et al. (2006)

Protein spot No.	Protein name	Expression level	NCBI accession number	Mol wt. (Da)	pI	Mascot score	Sequence coverage	No. of matched peptides	Function	References
D. New protein spots generated in [EMIM][OAc] and [BMIM][BF_4_]										
182,263	HSP70	New spot in response to both ILs	gi|14538021	70977	5.05	65	5%	2	Family of proteins that are produced by cells in response to exposure to stressful conditions	Mayer and Bukau (2005)
97	Catalase/peroxidase	New spot in response to both ILs	gi|367026346	82321	5.96	224	10%	5	Important enzyme in protecting the cell from oxidative damage by reactive oxygen species(ROS)	de Arruda et al. (2013)

*ND* not determined

The above results conclusively support the basis that ILs exert oxidative stress on *S. thermophile*, leading to metabolic regulation of many important pathways and anti-oxidative enzymes.

## Conclusion

The tolerance of industrially important strains such as *S. thermophile* towards green solvents like ILs has garnered new research interests. The study reports the effect of two imidazolium-based ionic liquids, [EMIM][OAc] and [BMIM][BF_4_] on the intracellular anti-oxidative enzymes activities as well as the proteome of *S. thermophile*. Global proteome analysis indicated IL-induced generation of oxidative stress, causing metabolic regulation of many important pathways and anti-oxidative enzymes. While on one hand, glycolysis, pentose phosphate pathway as well as ATP synthesis were found to be upregulated, on the other hand, TCA cycle and protein synthesis pathways were found to be retarded. An overall surge for NADPH production and reduction of ROS generation was revealed from the proteomic data. The study, thus, provides an important insight into the mechanism of action of imidazolium-based compatible and non-compatible ILs. It also adds to the emerging evidences of fungal adaptation responses and how such unique solvents can manipulate protein behavior.

## Electronic supplementary material

Below is the link to the electronic supplementary material.

**Supplementary Fig.** **1:** Network diagram of the pathways affected under the influence of ionic liquids in *Sporotrichum thermophile* (JPEG 374 kb)
