# Supplementary figures and images for: Proteomic profiling of Sporotrichum thermophile under the effect of ionic liquids: manifestation of an oxidative stress response
Source: 3 Biotech. 2019 May 30;9(6):240. doi: 10.1007/s13205-019-1771-z (PMC6542886; doi:10.1007/s13205-019-1771-z)

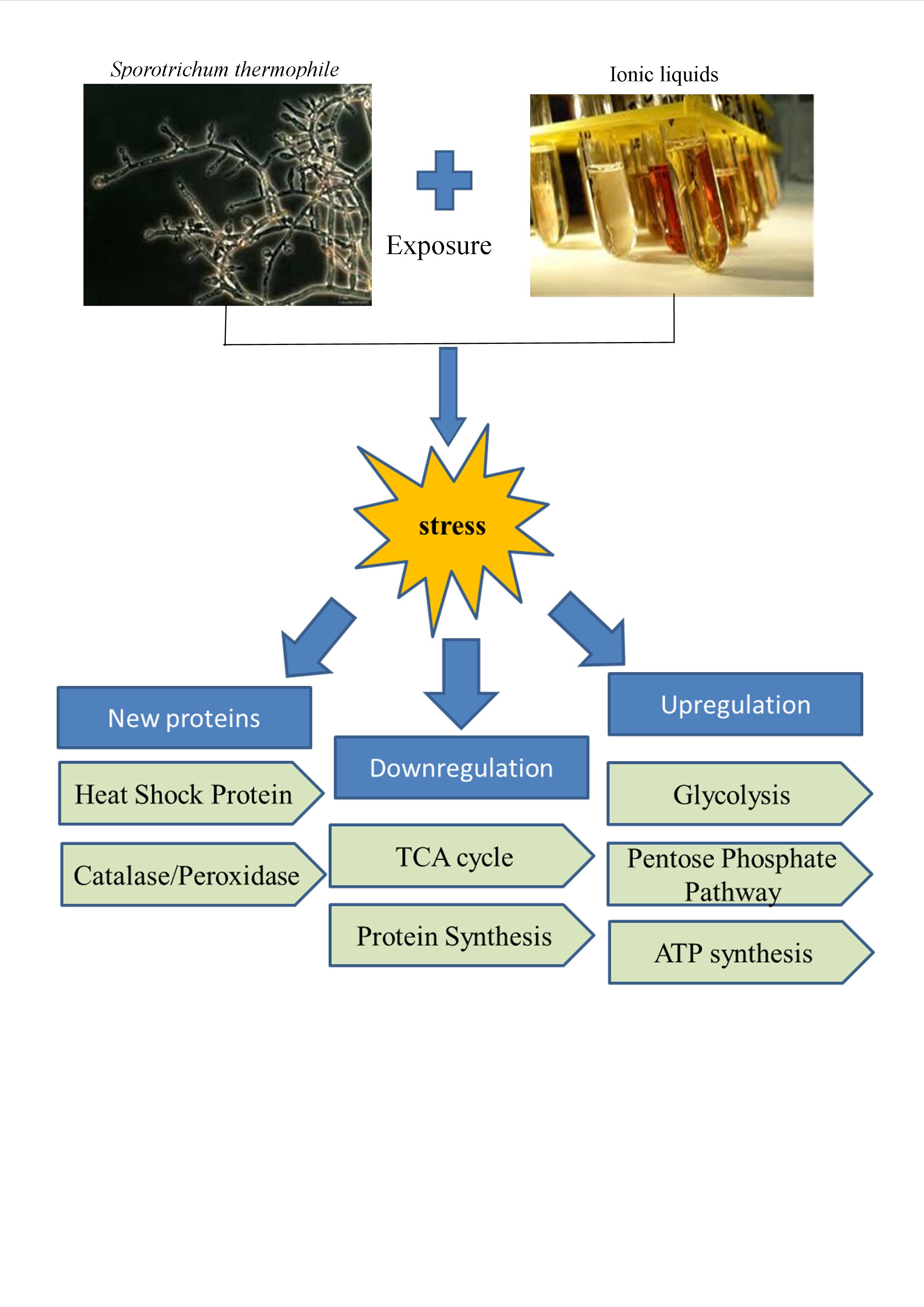

Supplement: Supplementary file 1 — Supplementary Fig. 1: Network diagram of the pathways affected under the influence of ionic liquids in Sporotrichum thermophile (JPEG 374 kb) [file 13205_2019_1771_MOESM1_ESM.jpg]
